# A versatile snap chip for high-density sub-nanoliter chip-to-chip reagent transfer

**DOI:** 10.1038/srep11688

**Published:** 2015-07-07

**Authors:** Huiyan Li, Jeffrey D. Munzar, Andy Ng, David Juncker

**Affiliations:** 1 Biomedical Engineering Department, McGill University, Montréal, QC, H3A 0G1, Canada; 2 McGill University and Genome Quebec Innovation Centre, McGill University, Montréal, QC, H3A 0G1, Canada; 3 Department of Neurology and Neurosurgery, McGill University, Montréal, QC, H3A 0G1, Canada

## Abstract

The coordinated delivery of minute amounts of different reagents is important for microfluidics and microarrays, but is dependent on advanced equipment such as microarrayers. Previously, we developed the snap chip for the direct transfer of reagents, thus realizing fluidic operations by only manipulating microscope slides. However, owing to the misalignment between arrays spotted on different slides, millimeter spacing was needed between spots and the array density was limited. In this work, we have developed a novel double transfer method and have transferred 625 spots cm^−2^, corresponding to >10000 spots for a standard microscope slide. A user-friendly snapping system was manufactured to make liquid handling straightforward. Misalignment, which for direct transfer ranged from 150–250 μm, was reduced to <40 μm for double transfer. The snap chip was used to quantify 50 proteins in 16 samples simultaneously, yielding limits of detection in the pg/mL range for 35 proteins. The versatility of the snap chip is illustrated with a 4-plex homogenous enzyme inhibition assay analyzing 128 conditions with precise timing. The versatility and high density of the snap chip with double transfer allows for the development of high throughput reagent transfer protocols compatible with a variety of applications.

Microarrays comprising a large number of spots, each with a different chemical or biochemical probe, allow for high-throughput biochemical assays to be performed using minute amounts of reagents^1^,^2^,^3^,^4^. However, the on-site delivery of reagents for microarray production requires expensive liquid handling robotics, such as inkjet or pin spotters, as well as skilled operators and maintenance. As such, most end users lack the technology to interface liquid reagents with individual microarray elements, limiting them to adding bulk samples to pre-made microarrays for analysis.

To address this, a number of platforms have been developed using liquid transfer technologies to deliver arrays of pre-spotted and stored reagents from one chip to another chip functioning as the assay substrate, thus avoiding the need for a microarray spotter during biochemical assays. These chip-to-chip transfer technologies contain self-aligned features on parallel surfaces that can be interfaced, with feature sizes ranging from those of standard 96- or 384-well plates to sub-microliter droplets. Chip-to-chip transfer methods can be categorized based on array density, as well as the combination of reagents that can be transferred between chips, for instance as either 1-reagent-to-N-reagent or as N-reagent-to-N-reagent transfer platforms.

For example, chip-to-chip transfers have been demonstrated by transferring reagents from gel droplets to cell monolayers in a 1-to-N manner^5^, or from gel droplets to cell-loaded gel droplets^6^ in an N-to-N manner, at array densities of 69 wells cm^−2^. This transfer method allowed various drug candidates to be screened against over one thousand individual cell cultures on a single chip.

Another option for chip-to-chip transfer is micropillar-microwell interfacing platforms, which allow for both N-to-1 and N-to-N transfers. For instance, Khademhosseini and colleagues developed micropillar arrays using PDMS^7^ or hydrogels^8^ loaded with various drugs, which could be inserted into microwell arrays containing cells at a pitch of 600 μm, achieving transfer densities of 278 wells cm^−2^. A microscope was used to align the two chips manually according to alignment features fabricated on each of the devices such that only a small number of spots were misaligned, however this also makes the device inconvenient for end-users and unpractical for point-of-care applications. The setup corresponded to an N-to-1 reagent transfer. Lee *et al.* spotted alginate solutions mixed with cells and gelated them on-chip to form micropillar arrays^9^. These could be inserted into complementary micro-wells filled with different drugs on a second chip at an array density of 49 wells cm^−2^ for screening in an N-to-N format. Similarly, in a more recent study, micro-wells were filled with cells, and different drugs laden on micro-pillars were inserted into the cell-loaded wells for incubation^10^. The pitch between wells was kept at 4.5 mm to be consistent with commercial 384-well plates, resulting in an array density of 4.9 wells cm^−2^.

Alternatively, an aperture-to-aperture transfer method relying on centrifugation has been developed by Kinoshita *et al.*^11^ to transfer liquid reagents between chips. On one chip, micro-wells fabricated from gel or plastic were loaded with ~0.5 μL of reagents in each well, and these reagent were transferred to an opposing chip by manually attaching the two chips face-to-face and then performing centrifugation. The micro-well array density achieved in this N-to-N transfer method was limited to 159 wells cm^−2^ due to well-to-well cross contamination during centrifugation.

In previous work, we developed a chip-to-chip reagent transfer technology, termed the snap chip, to transfer pre-spotted liquid droplets of reagents from microarray-to-microarray in an N-to-N manner, and used it to carry out multiplexed sandwich immunoassays^12^. Briefly, capture antibodies (cAbs) and detection antibodies (dAbs) were pre-spotted on an assay slide and a transfer slide, respectively, and stored in a freezer at −20 °C for up to 3 months. To carry out the assay, the two slides were retrieved, antigens were incubated on the assay slide, and the transfer and assay slides were snapped together, such that dAbs were transferred to the corresponding antigen-binding cAb spots on the assay slide. The platform allowed for immunoassays to be performed in an antibody colocalization microarray (ACM) format^13^, thereby overcoming cross-reactivity issues^14^,^15^,^16^,^17^,^18^,^19^,^20^. We also compared the double transfer snap chip immunoassay with commercial ELISA for two proteins, G-CSF and TNF-RI, using serum samples, and found that the correlation coefficient r was 0.94 for G-CSF and 0.82 for TNF-RI^21^. Furthermore, the end user could carry out the entire assay at the benchtop without the need for on-site spotting equipment. An array density of 100 spots cm^−2^ was achieved, limited by the alignment process and the imprecision of the inkjet spotter.

In summary, these previous efforts have highlighted the advantage of using chip-to-chip transfer. However, none of the previous techniques was truly microscopic without the aid of visual alignment features and a microscope, as spacing between adjacent spots, the main deterministic factor of array density and multiplexing capability, was typically limited to millimeters. Such limitation in array density was mainly caused by array production difficulties and alignment imprecision during the reagent transfer step.

In this work, we have developed a novel visualization-free miniaturization method to realize sub-nanoliter reagent transfer with micrometer-scale spacing between spots in an N-to-N manner, and improve the array density. Specifically, we have (i) re-designed the snap apparatus and demonstrated a lighter, polymer-based portable device without vacuum connections; (ii) developed a novel “double transfer” method significantly reducing the average misalignment from 164 μm to 16 μm while using a passive, optics-free alignment process, and achieved array densities up to 625 spots cm^−2^, which for a standard 25 × 75 mm^2^ microscope slide corresponds to >10000 spots when accounting for a 1.25 mm margin at the slide edge; (iii) visualized and quantified the misalignment distributions by direct and double transfer techniques using quiver plots to identify the source of the misalignment; (iv) demonstrated the multiplexing performance of the snap chip platform by simultaneously performing 16 independent 50-plex sandwich immunoassays on a slide, which to the best of our knowledge represents the largest multiplex sandwich immunoassay performed on a single microarray chip to date; (v) illustrated the versatility of the new snap chip platform with a proof-of-concept, 4-plex homogeneous enzymatic inhibition assay over a range of concentrations, and achieved comparable results with large volume experiments. These results illustrate the versatility of the snap chip and the power of miniaturization, which allows extracting more data while saving on sample, reagent cost, and time, and thus realizing the benefits of microarrays.

## Results and Discussion

### Simple-to-use snap apparatus

**Figure 1:**
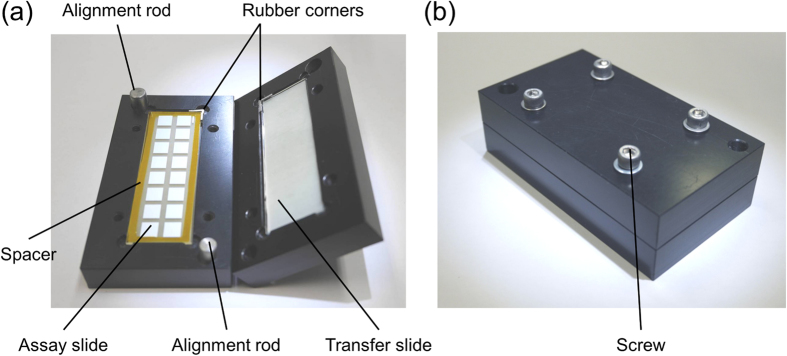
Photographs of the polymer snap apparatus. (**a**) Opened apparatus with a nitrocellulose assay slide and a 25 μm kapton polyimide film spacer placed around the 12 μm thick nitrocellulose pads (left) and an aminosilane transfer slide (right) in the chuck. A white silicone rubber cushion, visible behind both slides, allows pressure to be distributed evenly over the slide surface. A rubber corner is used in each chuck to secure the slide by pressing it against the opposite corner. (**b**) Photograph of a closed snap apparatus fastened with four screws.

The snapping of two slides with microscale features requires a dedicated apparatus with high alignment accuracy. We previously introduced an apparatus made of aluminum^12^. Although functional, this apparatus weighed 4.2 kg and required an on-site vacuum source to hold the slides in place, making it somewhat cumbersome to use. Here, we demonstrate a polymer snap apparatus that is more portable and user-friendly. The apparatus was machined out of polyoxymethylene, which has a high stiffness at room temperature and is often used for fabricating precision parts^22^. The polymeric snap apparatus weighs only 232 g and has a benchtop footprint of 9.8 × 5.5 cm^2^, as shown in Fig. 1. Two rods placed at opposing corners guide the slides together during snapping. Each slide is locked in place by a disposable rubber corner. The rubber corner is thinner than the microarray slide, such that it does not interfere with the snapping process. A thin silicone rubber cushion is then inserted between each slide and the apparatus, allowing for pressure to be evenly distributed over the slide surface during snapping. Finally, a constant gap between the slides is ensured by using a gasket spacer with a thickness chosen depending on the application, which is inserted between the two slides prior to snapping. The two halves of the apparatus are then snapped and held together by fastening screws, as shown in Fig. 1b, and reagent droplets from the transfer slide contact the assay slide. The use of a thin silicone rubber film and a spacer mitigated variations from operator-to-operator, and avoided the over-compression of droplets between slides. This improved polymer-based snap apparatus is simple-to-use and yields precise alignment.

### Double transfer method

**Figure 2:**
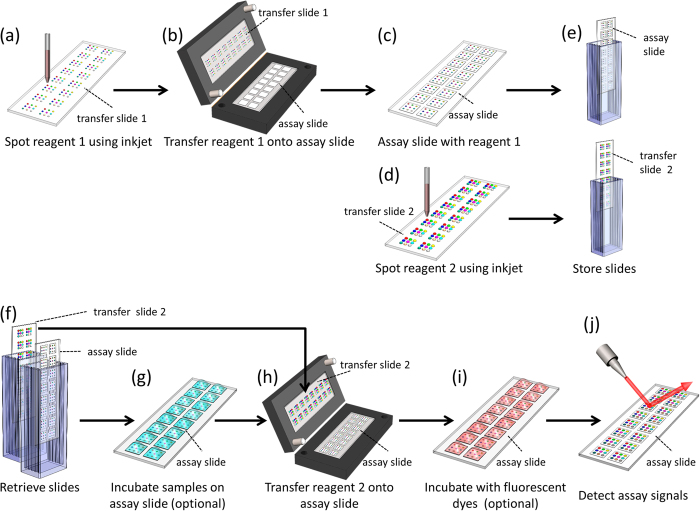
Schematic illustrating the process flow for snap chip fabrication using a double transfer protocol, as used for carrying out multiplexed biochemical assays. (**a**) Reagent 1 is spotted on transfer slide 1 and (**b**) transferred to the assay slide by snapping, (**c**) yielding a microarray of reagent 1 on the assay slide. (**d**) Reagent 2 is spotted on transfer slide 2 using an inkjet microarray spotter. (**e**) Both the assay slide and transfer slide 2 are stored for later use. (**f**) Prior to performing the assay, both slides are retrieved, and (**g**) the assay slide is incubated with a sample if necessary, then washed and dried. (**h**) The assay slide and transfer slide 2 are snapped together to transfer reagent 2 to the assay slide. Following rinsing, (**i**) the assay slide is incubated with fluorescent dyes, if necessary, and after another rinse, dried, and (**j**) the assay results are read out.

In this work, we have developed a novel double transfer method that is an improvement to the direct transfer process which we described previously^12^. The main difference between this protocol and the previous direct transfer technique is that here both reagent arrays are transferred onto the final assay slide. Previously, the first reagent (e.g. cAb) was directly spotted on the assay slide, while the second reagent (e.g. dAb) was spotted on the transfer slide in a mirror image configuration of the first reagent. The transfer slide was then directly interfaced with the assay slide. In the double transfer process, the first and the second reagents are spotted sequentially on two separate transfer slides with an inkjet spotter using the same spotting layout, such that both slides contained reagents patterned in the same layout and spotted at the same location on the spotter deck. Next, the microarray of reagent 1 is transferred to an empty assay slide using the snap apparatus. Finally, the microarray of reagent 2 (transferred spots) is interfaced with the assay slide (which comprises an array of sessile spots) using the snap apparatus. By aligning the two transfer slides to the same location on the inkjet spotter deck and to the same corner of the snap chip apparatus, the two reagents are consistently delivered to their respective positions on the assay slide. While it is possible to solve the misalignment problem by calibrating the inkjet spotter, it is in practice very difficult and cumbersome to do so due to regular day-to-day drift of the gantry system on the spotter which requires recalibration after each system initialization and after re-installation of the nozzle. Also, the double transfer method circumvents the need for a fiducial marker and camera recognition system compared to the direct transfer process; it also addresses angular misalignment as described in detail below. The process flow of the double transfer protocol, including fabrication of the snap chip, is illustrated in Fig. 2a–e.

Snap chip slides can be stored after spotting, as illustrated in Fig. 2e, allowing for slide production to be carried out ahead of time and potentially off-site, similar to the previous direct transfer method. Hygroscopic spotting additives, such as glycerol and betaine, are used in reagent solutions to prevent evaporation during spotting and to preserve the biological activity of the arrayed reagents during storage^23^,^24^. These spotting additives also allow for stored slides to be rehydrated prior to use by placing them in a humidity-saturated chamber.

### Characterization of misalignment

**Figure 3:**
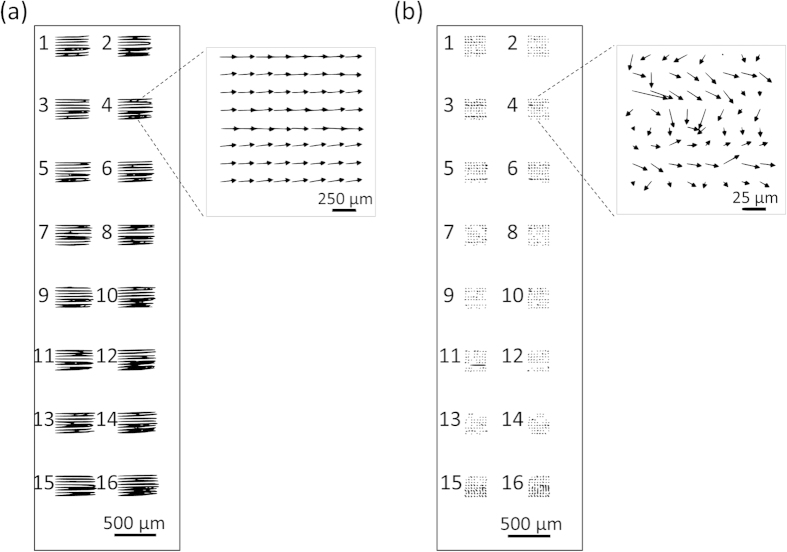
Direct and double transfer quiver plots of misalignment of 1024 spots in 16 arrays between the transferred and sessile drops. (**a**) Quiver plots of direct transfer shows systematic misalignment to the left as shown in the inset. (**b**) Double transfer misalignment is much reduced, and is across many different directions (see inset). Scale bars represent the magnitude of the misalignment for the quiver plot arrows.

**Figure 4:**
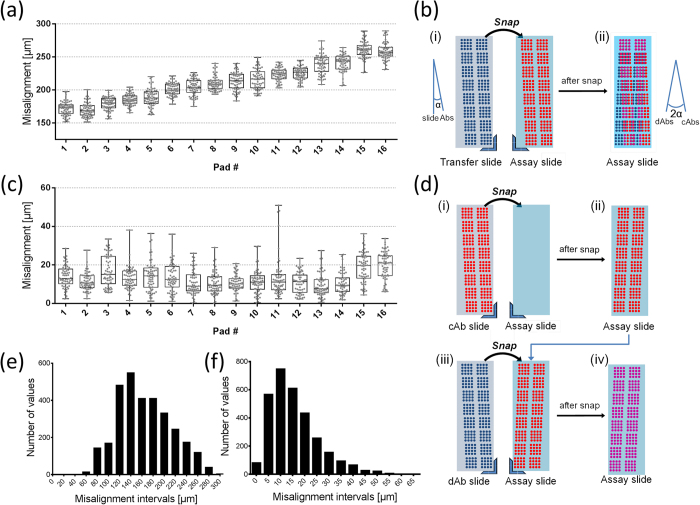
Misalignment of spots following direct and double transfer methods, with a schematic illustrating the consequences of angular misalignment. (**a**) Box plots of spatial misalignment in each of 16 nitrocellulose pads on one assay slide (1024 spots) using the direct transfer method. Nitrocellulose pad numbers are the same as indicated in Fig. 3(b) Schematic illustration on how mirroring of the transfer reagents on the assay slide amplifies the angular misalignment α between the slide and the inkjet XY stages by a factor 2. The box plots in (**a**) and (**c**) show the upper and lower quartile as box edges, the median value is indicated by the box centerline, and the whiskers show the range of the data. (**c**) Box plots showing the misalignment for spots of each of 16 nitrocellulose pads on one slide (1024 spots) using the double transfer method. (**d**) Schematic illustration of double transfer method to overcome the angular misalignment. (**e**,**f**) Distribution of misalignment of 3072 spots from three independent experiments: (**e**) the direct transfer method, with 20 μm bins, and (**f**) the double transfer method, with 5 μm bins.

Misalignment of the direct and double transfer methods was quantified by measuring the distance between pairs of overlaid spots of transferred and sessile drops. The misalignment was rendered visible by using fluorescently labeled antibodies as reagents; the sessile and transferred antibodies were labeled with green and red fluorescent dyes, respectively, Fig. 3. When using the direct transfer method, a systematic misalignment was observed between spots of first and second reagents along the horizontal axis, ranging from ~150 μm at the top of the slide to ~250 μm at the bottom, as shown in Fig. 4a and SI Fig. 2a-b.

When using the double transfer process, microarray misalignment was reduced to a few tens of micrometers in different orientations, appearing to be more random between the rows, as shown in Fig. 4c and SI Fig. 2c-d. In one of the three independent experiments we performed with 1024 spots, all spot pairs were within ~40 μm after the double transfer, with an average misalignment of 16 μm, except for 4 outlier spots on pad 11, Fig. 4c. An important advantage of the double transfer method is that the misalignment of the slide with the spotter is auto-compensated by the nature of the sequential transfer, Fig. 4d. The misalignment of the slide with the spotter is consistent when spotting on transfer slide 1 and transfer slide 2, because both slides were sequentially fixed to the same position on the spotter deck using the bottom right corner of the slides as an absolute reference point. As such, there is neither a requirement for absolute alignment, nor for an imaging system, as all alignment steps are relative to one another.

Distributions of misalignment in the form of spatial intervals from three independent experiments (3072 spots in total) are shown in Fig. 4e–f for direct and double transfer methods, respectively. The additional misalignment observed for direct transfer^12^ can thus be ascribed to two sources. Firstly, to the inaccuracy of the back-side alignment procedure that adds ~100 μm of error. Secondly, to the angular misalignment between slides and inkjet gantry that also adds ~100 μm of misalignment for the spots at the bottom of the slide. The alignment accuracy of the double transfer method is on average ten times better than direct transfer (164 μm *vs.* 16 μm). For the double transfer, 98% of the spots were within 41 μm, and the largest misalignment was 63 μm for all 3072 spots, as shown in Fig. 4f. The average misalignment is commensurate with the inconsistency of the inkjet spotter that was measured to be 6 μm on average, 33 μm maximum for 3072 single-printed spots. Thus, on average the misalignment is increased by 10 μm following a double transfer. The misalignment of the 4 outlier spots might be due to several reasons. During spotting, the inkjet nozzle operates about 1 mm above the substrate, and any contamination of the nozzle that might affect the droplet ejection angle by just a few degrees would lead to a misalignment of few tens of micrometers. Alternatively, inhomogeneity of the surface may pin droplets on one side, which may also result in misalignment. The observed pattern is indeed consistent with spurious effects, but not with misalignment due to the transfer alignment which would be expected to affect all spots in a systematic manner, as observed in the case of mirroring-based misalignment. The double transfer method is reliable, and arrays with 3136 antibody solutions, spotted in 16 arrays of 14 × 14 spots spaced at 450 μm, were routinely transferred with zero transfer failure and without cross-contamination between spots. (SI Fig. 1).

### 50-plex immunoassay using the snap chip

**Figure 5:**
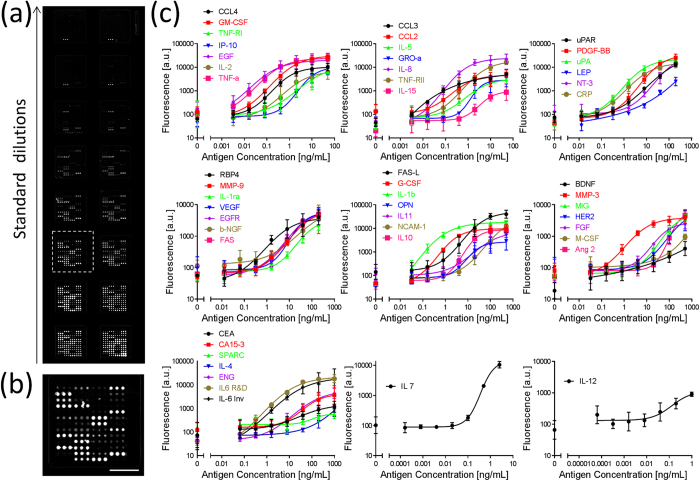
Fluorescent image of an assay slide and standard binding curves for 50 proteins simultaneously measured using the snap chip. (**a**) Fluorescent micrograph of a representative assay slide with 16 replicate arrays incubated in serial standard dilutions. (**b**) Close-up of the array within the dashed box in (**a**), with 153 spots and a 400 μm center-to-center spacing. Scale bar: 2 mm. (**c**) Standard curves for 50 proteins (for CA 15-3 the concentration is in unit/mL). Error bars represent standard deviations calculated from three independent experiments. Negative control fluorescence signals generated with 0 ng/mL protein concentrations are shown as separate data points.

To demonstrate the multiplexing capabilities of the snap chip using the double transfer method, an antibody colocalization microarray targeting breast cancer biomarkers^25^,^26^,^27^, cancer-related proteins, and cytokines^28^ was performed according to a protocol outlined in Fig. 2. The immunoassay consisted of a total of 50 different antibodies and GFP calibrations spots, spotted in triplicate per pad, resulting in 2448 spots per slide. Aminosilane functionalized slides were used as transfer slides for the cAbs and dAbs. These slides act as good transfer surfaces due to their moderate contact angle, which helps to widen spots and therefore facilitate alignment, while on the other hand resulting in high droplets extending tens of micrometers above the surface, so as to ensure contact with the assay slide. The assay slides were coated with ~12 μm thick nitrocellulose pads, which provided a large binding capacity for cAbs and also facilitated the subsequent transfer of dAb droplets by partially absorbing the liquid. After spotting and transferring the cAb, the assay slide and the transfer slide with dAbs were stored in a −20 °C freezer. To run an assay, the slides were first rehydrated as described in detail in the methods section. Seven serial dilutions of each protein and a negative control (no protein) were incubated in the eight wells of each column of nitrocellulose pads on the assay slide, Fig. 5a. Variations in spot sizes between triplicate spots for antibody pairs, which match across all array pads, can be attributed to volumetric errors in the inkjet spotting, depending on the spotted antibody, Fig. 5b. The variations in spot size did not affect immunoassay data analysis because of the homogeneity across all the pads, and might be mitigated with larger volumes for each spot. The fluorescence intensity was quantified, and standard curves were generated, and are grouped based on the binding ranges of each antibody pair and the signal intensity, Fig. 5c. The LODs of 35 proteins were found to be in the pg/mL range, with the lowest being 3.3 pg/mL for CCL3, SI Table 2. The LOD might be improved by further optimizing the assay and storage conditions, since the current ACM format allows the conditions for each spot to be optimized independently. Overall, this 50-plex sandwich immunoassay represents the highest multiplexing level of a cross-reactivity-free antibody microarray achieved to-date. Furthermore, colocalized reagent delivery allows the assay to incorporate multiple antibody pairs that would otherwise cross-react when mixed together, overcoming a major limitation of other multiplexed assay formats^29^, and therefore the number of proteins to be measured could be further expanded.

### Multiplex homogeneous enzymatic inhibition assays using the snap chip

Rapid and high throughput screening of potential drug leads against specific targets is an integral process of drug discovery and development^30^. As a demonstration of the versatility of the snap chip platform, we performed 128 simultaneous on-chip screening experiments by carrying out a proof-of-concept, homogeneous enzyme inhibition assay.

Human alpha thrombin, the central enzyme in hemostasis^31^, was selected as the target and four protease inhibitors (two potent thrombin inhibitors and two non-specific protease inhibitors) were tested with the snap chip using the double transfer method. The fast proteolytic activity of thrombin, on the order of minutes, represents a practical difficulty during bench top testing, as it requires precise temporal control of reagent delivery. When thrombin activity is assayed, for instance in a microtiter plate format, it is necessary to either carefully synchronize reagent delivery upon initiating the enzymatic reactions, or to use a multiplexed reagent delivery technology, such as multichannel pipettes or a multi-channel robotic system. To overcome manual reagent handling issues and minimize the volume of reagents needed, we carried out the assay using the snap chip, which allowed for nanoliter volume droplets containing thrombin to be interfaced with droplets containing inhibitor reagents and a fluorogenic substrate. This drastically reduced reagent volumes, while also allowing for simultaneous reagent delivery for all experimental conditions.

The four inhibitors were assayed at 6 dilutions, varying linearly from 0 to 25 nM, except for phenylmethanesulfonyl fluoride (PMSF), which was assayed from 5–25 μM. Multiple data points were extracted for each experimental condition. Two arrays of 64 droplets per slide, each containing three replicate droplets per inhibitor dilution condition (two for each PMSF condition), were imaged under the microscope. Three such slides were tested over the course of one week, generating 12–18 data points per experimental condition, for a total of 384 data points. For a negative control, a separate array of 6 droplets containing Argatroban and thrombin, but lacking a fluorogenic substrate, was included on the slide in a third array and generated baseline fluorescence.

Unlike previous snap chip assays that were conducted at the slide surface, a homogeneous assay was performed here. A fluorosilane monolayer was applied to the glass to form a non-reactive, non-absorbent surface, while at the same time ensuring the formation of hemispheric droplets for easy snap chip transfer. To carry out the assay, first droplets containing both an inhibitor and the fluorogenic substrate were spotted on transfer slide 1, and then transferred using the snap chip to the assay slide. Next, droplets containing thrombin were spotted on transfer slide 2, which was snapped together with the assay slide. After a short mixing time, the slides were unsnapped, resulting in two slides containing equal mixtures of thrombin, inhibitor and substrate (SI Fig. 3). The assay slide was imaged with a fluorescence microscope and the data was analyzed. Each slide contained an equal amount of reaction products, and as demonstrated here, transfer slide 2 could be used to generate a duplicate data point (SI Fig. 3) or stored as a backup aliquot, if desired.

**Figure 6:**
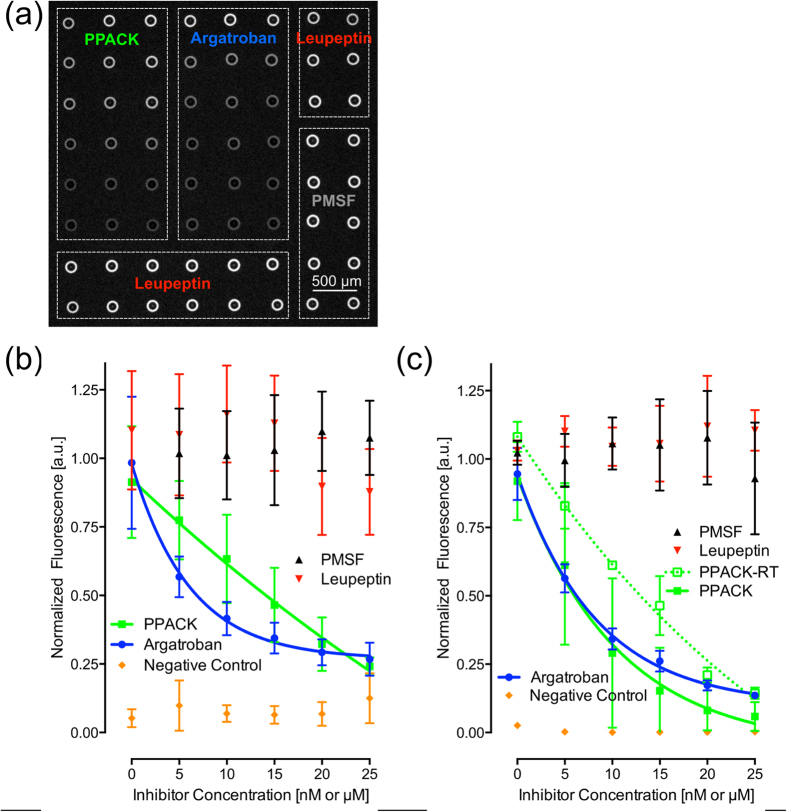
Fluorescence microscope image and results of multiplexed enzymatic inhibition assay of thrombin. (**a**) Fluorescence image obtained after 15 min of on-chip enzymatic activity. Experimental results and one phase exponential fits of inhibition assays conducted using (**b**) the snap chip or (**c**) conventional microcentrifuge tube assays; PPACK and Argatroban inhibit thrombin in a dose-dependent manner, while PMSF and Leupeptin do not, as expected (n = 3 independent experiments, error bars are standard deviation). The difference observed for the inhibition curves between the snap chip and microcentrifuge tube assays for PPACK is accounted for when the reagents in the microcentrifuge tube assay were stored at room temperature for 15 minutes prior to carrying out the assay (PPACK-RT).

The fluorescence generated from substrate turnover is shown in Fig. 6a, demonstrating the dose-dependent inhibition of thrombin within individual droplets. Note that the fluorescence image of the droplets appears as a torus due to a lensing effect of the hemispherical droplets under the microscope. Data analysis of the resulting fluorescence is shown in a normalized format in Fig. 6b. The results highlight the effective inhibition of human alpha-thrombin by the direct thrombin inhibitors D-phenylalanyl-L-prolyl-L-arginine chloromethyl ketone (PPACK) and Argatroban. Meanwhile, even at a 1000-fold higher concentration, PMSF showed no inhibitory activity against thrombin, with substrate turnover rates equivalent to that of Leupeptin, a non-targeting control for these experiments. Importantly, the results obtained with the snap chip are in good agreement with those obtained with microcentrifuge tubes that use 1000 times more sample, as shown in Fig. 6c. The data obtained with the snap chip and the benchmark, large volume microcentrifuge assays for Argatroban are in excellent agreement, generating the same dose-specific inhibition of thrombin over the range of inhibitor concentrations tested. However, for PPACK, the dose-dependent thrombin inhibition in the snap chip assay indicated decreased inhibitory activity. It is known from the literature that the chloromethyl ketone moiety is hydrolyzed in basic solutions^32^,^33^, and the assay was carried out at a pH of 7.5, which could lead to significant hydrolysis. The reagents used in the large-scale microcentrifuge tube assay were kept on ice until initiation of the reaction, hence hydrolysis would be expected to be slowed, whereas for the nanoscale snap chip assays, printing and chip handling were carried out at room temperature, potentially leading to a more pronounced inhibitor hydrolysis. To assess this hypothesis, we performed a large volume assay in microcentrifuge tubes with pre-incubation of PPACK and substrate at room temperature (PPACK-RT) for 15 min prior to mixing with thrombin, Fig. 6c. When following the same temperature cycles, the inhibition curves of the snap chip and microcentrifuge assays show the same kinetics. Argatroban is not prone to hydrolysis under these conditions, further corroborating this analysis. In summary, these results illustrate that the snap chip can be used in place of traditional large-volume methods to screen candidate inhibitors against targets of interest in a high throughput format. However, if quantitative results are desired, care must be taken to compensate for secondary effects such as temperature or oxygen sensitivity that may skew the results obtained with the snap chip.

## Conclusion

In this work, we have developed a novel, double-transfer method, which makes visualization-free microscale chip-to-chip transfer feasible, allowing for the transfer of thousands of sub-nanoliter reagents in parallel with a 400 μm center-to-center spacing, corresponding to an unprecedented density of 625 spots cm^−2^ from chip to chip in an N-to-N manner with improved versatility. The alignment accuracy of >98% of spots is <40 μm. A new snap apparatus was developed to make this technology more convenient and compatible for non-expert end users, both at the bench top and for use in point-of-care analysis. As an example of the high throughput capabilities of the snap chip, a 50-plex immunoassay was carried out, representing the largest cross-reactivity-free multiplex sandwich antibody microarray demonstrated to date, achieving pg/mL sensitivity for antigens with high affinity antibodies. The versatility of the snap chip was also demonstrated with a high-throughput enzymatic inhibition screening assay performed using 4 nL aliquots, yielding results consistent with a microliter-volume assay benchmark, but with 1000-fold lower reagent volumes.

The snap chip is a generic platform that provides a technological solution for interfacing liquid reagents with microarray elements in a high throughput manner, as demonstrated here. Thus far, the snap chip has been used only to “copy” a single aliquot of liquid droplets between two microarray slides, but it could be expanded to perform more complex operations. The spacing between spots may be reduced further, for example by using smaller droplets or by making the surfaces more hydrophobic and thus the droplets more rounded; if center-to-center spacing of 200 μm could be reached, >40000 droplets could be manipulated using a standard microscope slide. In this work a single inkjet nozzle was used. For larger scale arrays, high speed microarrayers can be used to speed up the chip fabrication, and importantly, snap chip technology allows for chip fabrication and assay experimentation to be uncoupled. Snap chips could also be used for work with living organisms, such as bacteria or cells, and might for example be developed for the analysis of single cell content and excretions^34^. The collective transfer of thousands of reagents will make the snap chip attractive to other applications that would benefit from the high throughput manipulation of minute reagent volumes.

## Methods

### Materials

Goat anti-mouse IgG (H + L) labeled with Alexa Fluor 532 and chicken anti-goat IgG (H + L) labeled with Alexa Fluor 647 were purchased from Life Technologies. Cognate antibody pairs and proteins used are listed in SI Table 1. Streptavidin-conjugated Cy5 was obtained from Rockland. Phosphate buffered saline (PBS) tablets and glass slides were purchased from Fisher Scientific. Glycerol, 1,3-butanediol, betaine, trichloro(1H,1H,2H,2H-perfluorooctyl)silane, acetone, hydrochloric acid, Tween-20, argatroban monohydrate, phenylmethanesulfonyl fluoride (PMSF) and leupeptin hydrochloride were purchased from Sigma-Aldrich. Bovine serum albumin (BSA) was purchased from Jackson ImmunoResearch Laboratories, Inc. BSA-free StabilGuard Choice Microarray Stabilizer was purchased from SurModics, Inc. Human alpha-thrombin (HCT-0020), fluorogenic substrate (SN20) and the inhibitor D-phenylalanyl-L-prolyl-L-arginine chloromethyl ketone (PPACK) were purchased from Haematologic Technologies Inc. Nitrocellulose coated slides were purchased from Grace Bio-Laboratories, and aminosilane coated slides were purchased from Schott North America.

### Characterization of misalignment

For both direct and double transfer methods, Alexa 532 labeled goat anti-mouse IgGs were used as the capture antibody (cAb) and Alexa 647 labeled chicken anti-goat IgGs were used as the detection antibody (dAb). Each experiment was repeated 3 times independently, and each time 1024 duplicated spots (64 spots per nitrocellulose pad) were analyzed per slide. The direct transfer method, which uses a back-side alignment mark, was described in detail in our previous work^12^. Briefly, goat anti-mouse IgGs were spotted on a nitrocellulose slide and chicken anti-goat IgGs were spotted on an aminosilane slide starting on the top of the alignment mark, then the two slides were fixed against the corners of the slide chuck and snapped together to transfer the antibodies. In the double transfer method, goat anti-mouse IgGs and chicken anti-goat IgGs were spotted on separate aminosilane slides and then sequentially transferred to the same nitrocellulose slide. The center-to-center distances between each primary and secondary antibody spot were measured using Image J software, and the Matplotlib library was used to make quiver plots^35^.

### 50-plex sandwich immunoassays using the snap chip

The concentrations of all the cAbs and dAbs used in this work are listed in SI Table 1. An inkjet spotter (Nanoplotter 2.0, GeSiM) was used to spot antibody microarrays at a relative humidity of 80%. The center-to-center distance between spots was 400 μm, and 2448 spots were spotted on a single slide. dAbs were spotted on an aminosilane coated slide (transfer slide 2) in PBS that contained 20% glycerol and 1% BSA, and each spot was 0.8 nL in volume. cAbs were spotted on another aminosilane coated slide (transfer slide 1) in PBS buffer containing 20% glycerol and 1% BSA, with a 0.4 nL volume for each spot. The transfer slide with cAbs was snapped together with a nitrocellulose slide (assay slide) in the snap apparatus for 1 min and then separated. GFP antibodies were also spotted to each of the 16 nitrocellulose pads to measure GFP antigens with the same concentration for data normalization across the whole slide. After overnight incubation, the assay slide was clamped with a slide module gasket (Grace Bio-Laboratories, Inc.) to separate it into 16 compartments. Next, the assay slide was washed 3 times with PBS containing 0.1% Tween-20 (PBST) on the shaker at 450 rpm for 5 min, followed by blocking with StabilGuard for 1 h on the shaker at 450 rpm, and finally the slide was dried under a stream of nitrogen. The blocked assay slide and the dAb transfer slide, which encompass 50 antibody pairs, were stored in individual airtight bags with desiccant in a −20 °C freezer.

To carry out the immunoassay, the assay slide was retrieved from the freezer and kept at room temperature for 30 min before the bag was opened. To prepare 7-point serial diluted sample solutions, all 50 proteins were spiked in PBS containing 0.05% Tween-20 with a five-fold dilution factor; the starting concentrations of which are listed in SI Table 1. The assay slide was clamped with a 16-compartment slide module gasket to separate the whole slide into 16 wells for sample incubation. The 7 protein dilution solutions, together with a protein-free blank, were incubated on the slide at 4 °C on the shaker at 450 rpm overnight. Subsequently, the gasket was removed and the slide was rinsed 3 times with PBST on the shaker at 450 rpm for 5 min, followed by brief drying using nitrogen gas.

To apply dAbs, the transfer slide with dAbs was retrieved from the freezer and kept for 30 min at room temperature before the bag was opened, and then incubated in a humidified petri dish for 20 min to rehydrate the glycerol droplets containing antibodies. The rehydrated dAb transfer slide and the assay slide were then fixed into the slide chucks of the snap apparatus, with a rubber cushion on the back of each slide and a 25 μm thick kapton spacer attached to the assay slide to allow for correct droplet bridging between the slides. The slides were snapped together, and the assembly was secured by screws and kept closed for 1 min. Next, the two slides were separated and the assay slide was incubated in a humidity-saturated petri dish for 1 h. A 16-compartment gasket was then clamped on the assay slide, and each well was rinsed 4 times using PBST on the shaker at 450 rpm for 5 min and subsequently incubated with 2.5 μg/mL streptavidin-Cy5 solutions prepared in PBS for 20 min on the shaker at 450 rpm. After rinsing 3 times using PBST and once with distilled water, the slide was dried under a stream of nitrogen and scanned using a fluorescence microarray scanner.

### Multiplex enzymatic inhibition assay using the snap chip

Fluorosilane-coated glass slides were fabricated using a cleaning^36^ and vapor deposition method^37^ with slight modifications. Briefly, glass slides were washed thoroughly with water and then dipped in 2 M hydrochloric acid for 5 min with sonication. Slides were washed once more in water, then in acetone, and then dried using a nitrogen stream. The slides were further cleaned and activated by plasma treatment for 1 min (PlasmaEtch PE-50, PlasmaEtch), followed by vapor deposition of trichloro(1H,1H,2H,2H-perfluorooctyl)silane in a desiccator for 30 min. Contact angles of 1 μL water droplets on the coated slides was 104 degrees as measured using a contact angle goniometer (VCA Optima, AST Products Inc).

Dilutions of inhibitors with a constant substrate concentration were made in a 384 well plate using a hygroscopic buffer, consisting of 1 M betaine and 12.5% 1,3-butanediol in PBS, which minimized evaporation during spotting and handling of the snap chips. The substrate was mixed to a concentration of 5 μM for all inhibitor conditions. For PPACK, Argatroban and Leupeptin, inhibitor concentration was varied linearly from 0 to 50 nM, and for PMSF from 10 to 50 μM. Thrombin was diluted in the same hygroscopic buffer to a concentration of 20 nM, and all reagents were kept on ice in a 384-well plate until transferred to the spotting environmental chamber, and spotting took 15 minutes.

8 nL droplets of the mixture containing inhibitor and substrate were arrayed on the fluorosilane-coated transfer slide 1 using the inkjet spotter at a pitch of 475 μm in 8 by 8 droplet arrays at 80% humidity and 25 °C. Each 64-droplet array contained two or three replicates of each condition, and two such arrays were spotted per slide. After spotting, the inhibitor/substrate droplet array was snapped together with a second fluorosilane-coated assay slide using the snap apparatus with a 62.5 μm thick Scotch tape spacer and 1 min contact time. After unsnapping, the assay slide retained half of the droplet volume (4 nL) of inhibitor/substrate.

The thrombin mixture was spotted onto a third fluorosilane-coated slide at the same pitch, but using only 4 nL of reagent per droplet. This array was immediately snapped against the assay slide containing the copied inhibitor/substrate array. Droplet-to-droplet contact was maintained for 2 min in the snap chip apparatus, allowing for reagents to mix and diffuse across slides, after which the two slides were separated and both were incubated at 25 °C and 80% relative humidity for 15 min. During this time, thrombin cleaved the fluorogenic substrate within the droplets on both slides, leading to fluorescence generation. Effective concentrations of reagents in the final 4 nL droplet microarrays were 2.5 μM substrate, 10 nM thrombin, and from 0 to 25 nM inhibitor (5–25 μM for PMSF).

### Conventional enzymatic inhibition assay with microliter volumes

To provide a benchmark for the performance of the snap chip-scale enzymatic inhibition assay, the activities of the thrombin inhibitors were tested for the same dilution levels and with the same substrate and thrombin concentration, but with a total volume of 20 μL in conventional microcentrifuge tubes. Benchmark assays were carried out in three independent experiments. All reagents were kept on ice until initiation of the reaction, except for the room-temperature PPACK inhibition test, whereby reagents were kept at 25 °C for 15 minutes prior to initiation. Initiation consisted of adding thrombin to the substrate-inhibitor mixture. After initiation, the assay was incubated for 15 min at 25 °C, after which the fluorogenic substrate turnover was quantified using a fluorospectrometer (Nanodrop 3300, Thermo Scientific) at an emission wavelength of 470 nm.

### Imaging and data analysis

A fluorescence microarray scanner (Axon GenePix 4000B) with 532 nm and 635 nm lasers were used to scan the capture and detection antibody spots, respectively, for misalignment characterization, and the 635 nm laser was used to image sandwich immunoassays. The net intensity of each spot was extracted using Array-Pro Analyzer (MediaCybernetics) software, which included subtracting background signal around each spot from the raw intensity. The LOD for each protein in the immunoassay was calculated using GraphPad Prism (GraphPad Software), and was defined as the Y-intercept of the standard curve incremented by three times the standard deviation of three independent assays.

To obtain enzymatic inhibition assay signals, droplets on the fluorosilane assay slide were imaged using a CCD camera (QuantEM 512SC, Photometrics) mounted on an inverted fluorescence microscope (TE-2000-E, Nikon) with an ultraviolet filter (UV-2A, Nikon) with excitation wavelengths of 330–380 nm, detection above 420 nm. Images were corrected for background (acquired from a blank slide) and analyzed using a custom script (MATLAB, Mathworks) in order to calculate the average intensity of the torus of fluorescence generated for each droplet in the array. Three separate experiments carried out on non-consecutive days over the course of one week using fresh reagents were conducted to assess assay reproducibility.

## Additional Information

**How to cite this article**: Li, H. *et al.* A versatile snap chip for high-density sub-nanoliter chip-to-chip reagent transfer. *Sci. Rep.* **5**, 11688; doi: 10.1038/srep11688 (2015).

## Supplementary Material

Supplementary Information
