# Supplementary material for: A versatile snap chip for high-density sub-nanoliter chip-to-chip reagent transfer
Source: Sci Rep. 2015 Jul 7;5:11688. doi: 10.1038/srep11688 (PMC4493572; doi:10.1038/srep11688)
Supplement: Supplementary Information [file srep11688-s1.pdf]

Supplementary Information

A versatile snap chip for high-density sub-nanoliter chip-to-chip reagent transfer

Huiyan Li[a,b], Jeffrey D. Munzar[a,b], Andy Ng[a,b], and David Juncker[a,b,c,*]

[a]Biomedical Engineering Department, [b]McGill University and Genome Quebec Innovation Centre, [c]Department of Neurology and Neurosurgery, McGill University, Montréal, QC, H3A 0G1, Canada

*To whom correspondence should be addressed. E-mail：david.juncker@mcgill.ca

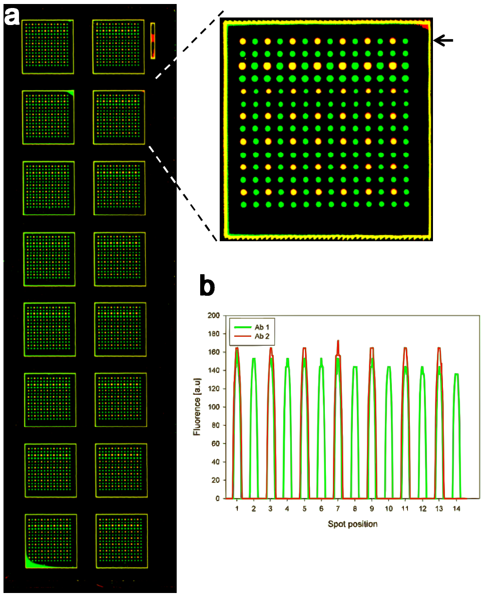

SI Figure 1: (a) Scan of the snap chip assay slide using 532 nm and 633 nm lasers after transferring 3136 antibody pairs for alignment quantification. Alexa 532 labeled goat IgG was chosen as the cAbs, and Alexa 633 labeled anti-goat IgG was spotted on every second spot on the dAb transfer slide and transferred to the assay slide. The center-to-center spacing between spots was 450 µm. Variations in spot size between rows, which match across all of the array pads, can be attributed to volumetric errors in the inkjet spotting, which was done two rows at a time. (b) Fluorescence intensity profiles of the green and red protein spots in the first row, as indicated by the arrow in the inset.

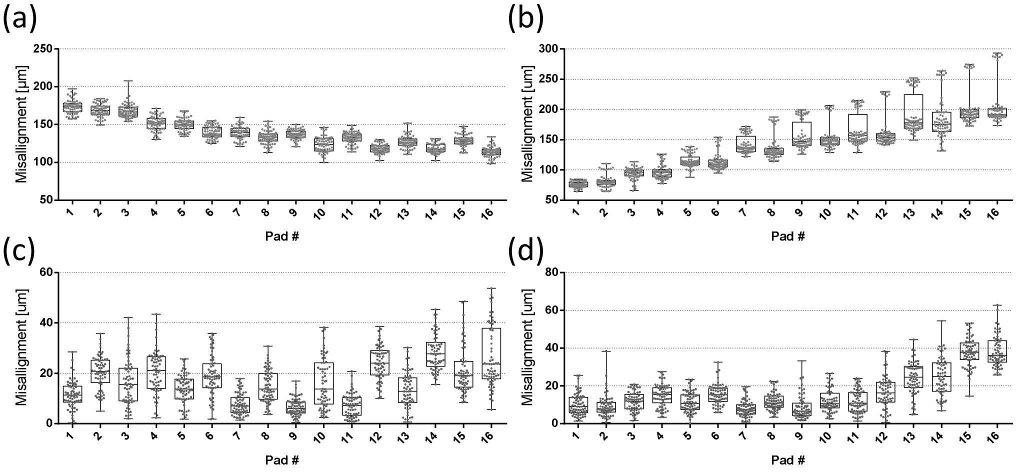

SI Figure 2: Box plots of spatial misalignment in each of 16 nitrocellulose pads of individual assay slides for the additional experiments using the direct transfer (a-b) and double transfer (c-d) method. Nitrocellulose pad numbers are the same as indicated in Fig. 3. The trends in (a,b) reflect the mirror misalignment, while in (c,d) misalignment doesn't show a systematic trend. There seems to be a trend for increased misalignment on pads 15 & 16 for the double transfer method, but more experiments are needed to uncover the source of it.

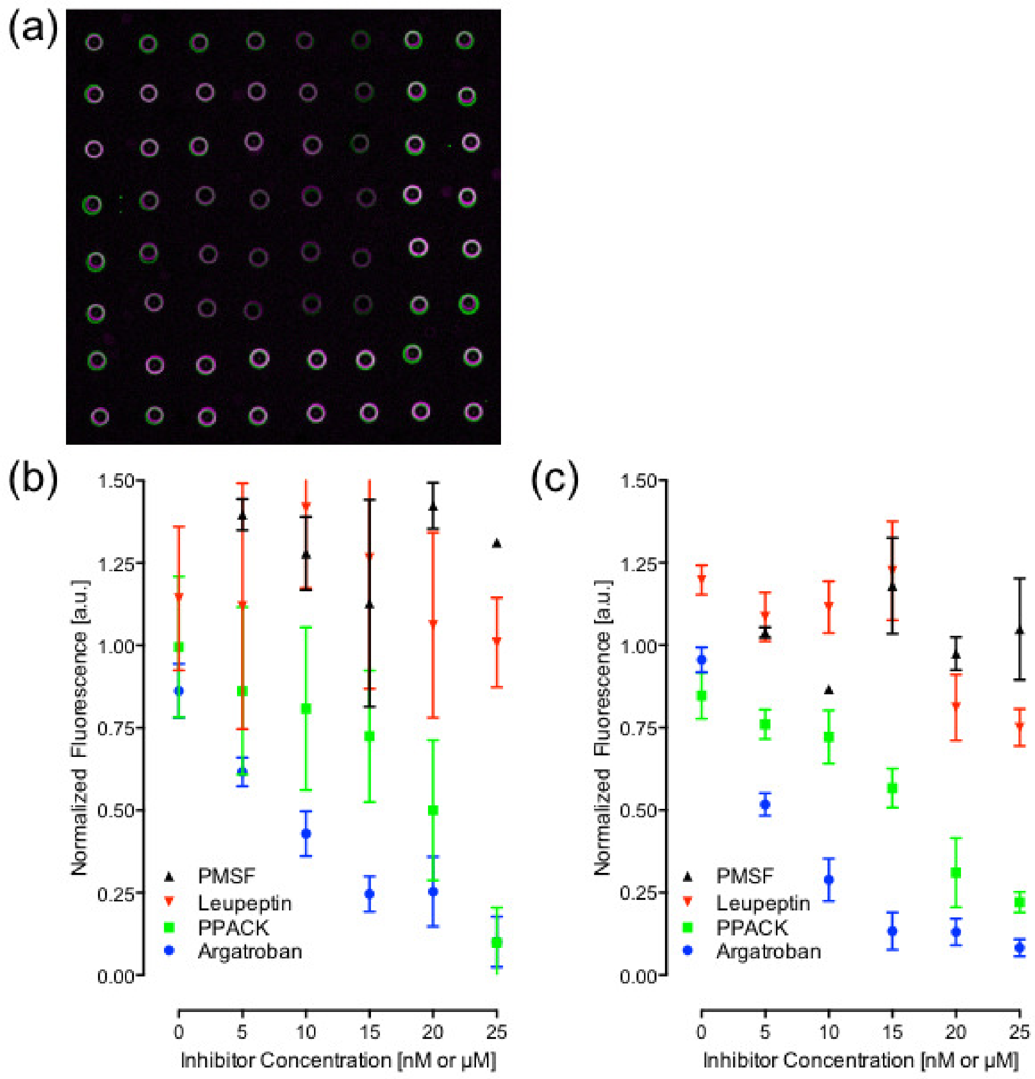

SI Figure 3: (a) Overlay of fluorescence images of assay (magenta) and transfer (green) slides used in the enzymatic assay experiments, showing equal partitioning of droplets between slides after snapping. Comparison of the analysis of enzymatic inhibition assay signals obtained when imaging the (b) assay slide, and (c) the replicate transfer slide. Error bars represent 1 SD of the data from a single experiment (N=3, except for PMSF: N=2). The deviations in the inhibition of thrombin between assay and transfer slides are within the experimental error observed between experiments carried out on separate days.

SI Table 1 Names, suppliers and concentrations of proteins and antibodies used in this study.

Name	Abbr.	cAb	Antigen	dAb	Supplier	cAb (μg/ml)	Starting[Ag] (ng/ml)	dAb (μg/ml)
Angiopoietin-2	ANG2	MAB098	623-AN	BAM0981	R&D	400	500	50
Brain-derived neurotrophic factor	BDNF	MAB848	248-BD	BAM648	R&D	400	500	50
Cancer antigen 15-3	CA 15-3	10-CA15A	C0050-21 US Biological	10-CA15B	Fitzgerald	400	1 (U/ml)	100
Carcinoembryonic antigen	CEA	C1300-02X	C1300-08E	C1300-07A	US Biological	400	1000	108
Chemokine (C-X-C motif) ligand 10	CXCL 10/IP-10	MAB266	266-IP	BAF266	R&D	400	50	50
C-reactive protein	CRP	MAB17071	1707-CR	BAM17072	R&D	400	200	50
Endoglin	ENG	MAB10972	1097-EN	BAF1097	R&D	400	1000	100
Epidermal growth factor	EGF	MAB636	236-EG	BAF236	R&D	400	50	50
Epidermal growth factor receptor	EGFR	AF231	1095-ER	BAF231	R&D	400	200	50
FAS ligand	FAS-L	MAB126	126-FL	BAF126	R&D	400	500	50
Fibroblast growth factor	FGF	MAB233	233-FB	BAM233	R&D	400	500	50
Green fluorescent protein	GFP	600-301-215	000-001-215	600-106-215	Rockland Immuno-chemical	400	20	50

Granulocyte colony-stimulating factor	G-CSF	MAB214	214-CS	BAF214	R&D	400	500	50
Granulocyte-macrophage colony-stimulating factor	GM-CSF	MAB615	215-GM	BAM215	R&D	400	50	50
Growth-related oncogene alpha	GRO-α	MAB275	275-GR	BAF275	R&D	400	50	50
Human epidermal growth factor receptor 2	HER2	MAB1129	1129ER	BAF1129	R&D	400	500	100
Human platelet-derived growth factor BB	PDGF-BB	385-PR/ carrier free	220-BB	BAF220	R&D	400	200	50
Interleukin 1 beta	IL-1β	MAB601	201-LB	BAF201	R&D	400	500	50
Interleukin 1 receptor antagonist	IL-1ra	MAB280	280-RA	BAF280	R&D	400	200	50
Interleukin 15	IL-15	MAB647	247-IL	BAM247	R&D	400	50	50
Interleukin 12	IL-12	CHC1563	CHC1563	CHC1563	Invitrogen	400	1	50
Interleukin 11	IL-11	MAB618	218-IL	BAF218	R&D	400	500	50
Interleukin 10	IL-10	CHC1323	CHC1323	CHC1323	Invitrogen	400	500	50
Interleukin 8	IL-8	CHC1303	CHC1303	CHC1303	Invitrogen	400	50	50
Interleukin 7	IL-7	MAB207	207-IL	BAF207	R&D	400	2.5	50
Interleukin 6	IL-6	MAB206	206-IL	BAF206	R&D	400	1000	50
Interleukin 6	IL-6	CHC1263	CHC1263	CHC1263	Invitrogen	400	1	50
Interleukin 5	IL-5	MAB405	205-IL	BAM6051	R&D	400	50	50
Interleukin 4	IL-4	MAB604	204-IL	BAF204	R&D	400	1000	50
Interleukin 2	IL-2	MAB602	202-IL	BAF202	R&D	400	50	50
Leptin	LEP	MAB398	398-LP	BAM398	R&D	400	200	50
Monokine induced by interferon-gamma	MIG	MAB392	392-MG	BAF392	R&D	400	500	50
Macrophage	CCL3	AF-270-	270-LD	BAF270	R&D	400	50	50

inflammatory protein 1 alpha	/ MIP-1α	NA						
Macrophage inflammatory protein 1 beta	CCL4 / MIP-1β	MAB271	271-BME	BAF271	R&D	400	50	50
Matrix metalloproteinase 3	MMP-3	AF513	513-MP	BAF513	R&D	160	500	50
Macrophage colony-stimulating factor	M-CSF	MAB616	216-MC-005	BAF216	R&D	400	500	50
Matrix metalloproteinase 9	MMP-9	MAB936	911-MP	BAF911	R&D	400	200	50
Monocyte chemotactic protein1	CCL2 /MCP -1	MAB679	279-MC	BAF279	R&D	400	50	50
Neural cell adhesion molecule 1	NCAM-1	MAB2408	2408-NC	BAF2408	R&D	400	500	50
Nerve growth factor (beta polypeptide)	β-NGF	MAB256	256-GF	BAF256	R&D	400	200	50
Neurotrophin-3	NT-3	MAB267	267-N3	BAF267	R&D	400	200	50
Osteopontin	OPN	MAB14332	1433-OP	BAF1433	R&D	200	500	50
Retinol binding protein 4	RBP4	MAB33781	3378-LC	BAM33782	R&D	400	200	50
Secreted protein acidic and rich in cysteine	SPARC	MAB941	941-SP	BAF941	R&D	400	1000	100
Tumor necrosis factor alpha	TNF-α	MAB610	210-TA	BAF210	R&D	400	50	50
Tumor necrosis factor receptor 1	TNF-RI	MAB625	636-RI	BAF225	R&D	400	50	50
Tumor necrosis factor receptor II	TNF-RII	MAB726	1089-R2	BAF726	R&D	400	50	50
Tumor necrosis factor receptor	FAS/TNFRS	MAB144	326-FS	BAF326	R&D	400	200	50

superfamily 6	F6							
u-Plasminogen Activator/Urokinase	uPA	MAB1310	1310-SE	BAF1310	R&D	400	200	50
u-Plasminogen Activator/Urokinase receptor	uPAR (CD87)	MAB807	807-UK	BAF807	R&D	400	200	50
Vascular endothelial growth factor	VEGF	MAB293	293-VE	BAF293	R&D	400	200	50

SI Table 2 Limits of detection (LODs) for the 50 proteins spiked in buffer, calculated from the results of 3 independent experiments. The LOD for CA 15-3 is in U/ml (*). [a]R&D Systems, [b]Invitrogen.

Protein name	LOD (pg/ml)	Protein name	LOD (pg/ml)
ANG2	13009.3	IL-6[b]	2137.2
BDNF	100.2	IL-5	76.7
CA 15-3*	4896.7	IL-4	14545.1
CEA	5385.4	IL-2	75.6
CXCL10/IP-10	167.7	LEP	402
CRP	44.1	MIG	1050.8
ENG	619.8	CCL3/MIP-1α	3.3
EGF	183.6	CCL4/MIP-1β	12.1
EGFR	188.1	MMP-3	99.9
FAS-L	446.2	M-CSF	8242.8
FGF	1032.7	MMP-9	310.8
G-CSF	39.2	CCL2/MCP-1	54.7

GM-CSF	3.8	NCAM-1	1694.3
GRO-α	300.3	B-NGF	1388.8
HER2	3491.3	NT-3	580.8
PDGF-BB	73.1	OPN	1908.5
IL-1β	787.7	RBP4	116.3
IL-1ra	1076.8	SPARC	45883
IL-15	823.5	TNF-α	4.4
IL-12	30.3	TNF-RI	129.8
IL-11	1212.9	TNF-RII	12
IL-10	610.3	FAS/TNFRSF6	276.6
IL-8	6.6	uPA	23.9
IL-7	25.9	uPAR(CD87)	75.5
IL-6[a]	83.6	VEGF	673.7
